# Supplementary figures and images for: Cellular Prion Protein Expression in the Brain Tissue from Brucella ceti-Infected Striped Dolphins (Stenella coeruleoalba)
Source: Animals (Basel). 2022 May 19;12(10):1304. doi: 10.3390/ani12101304 (PMC9137499; doi:10.3390/ani12101304)

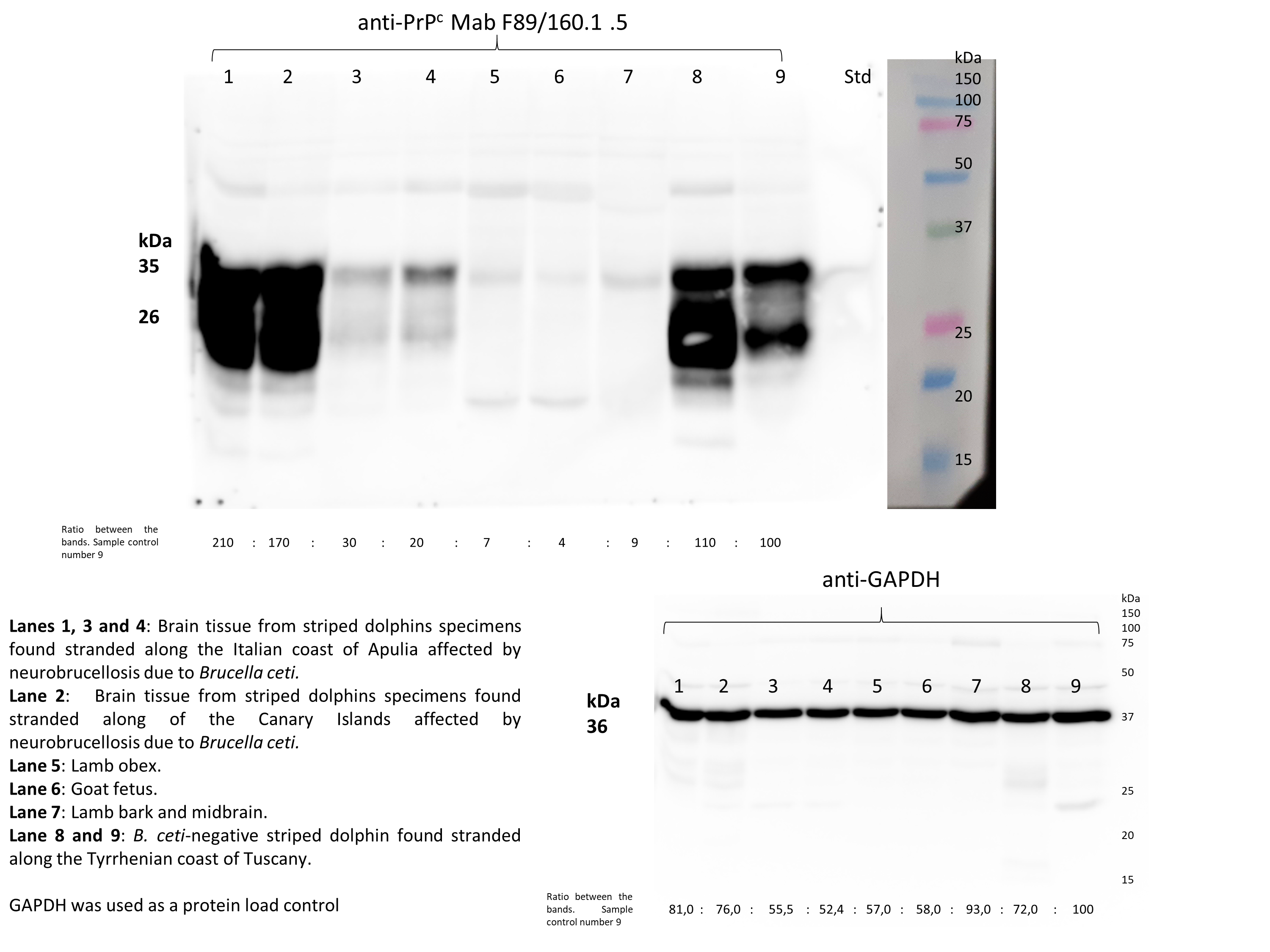

Supplement: Supplementary file 1 [file animals-12-01304-s001.zip › FigureS1.TIF]

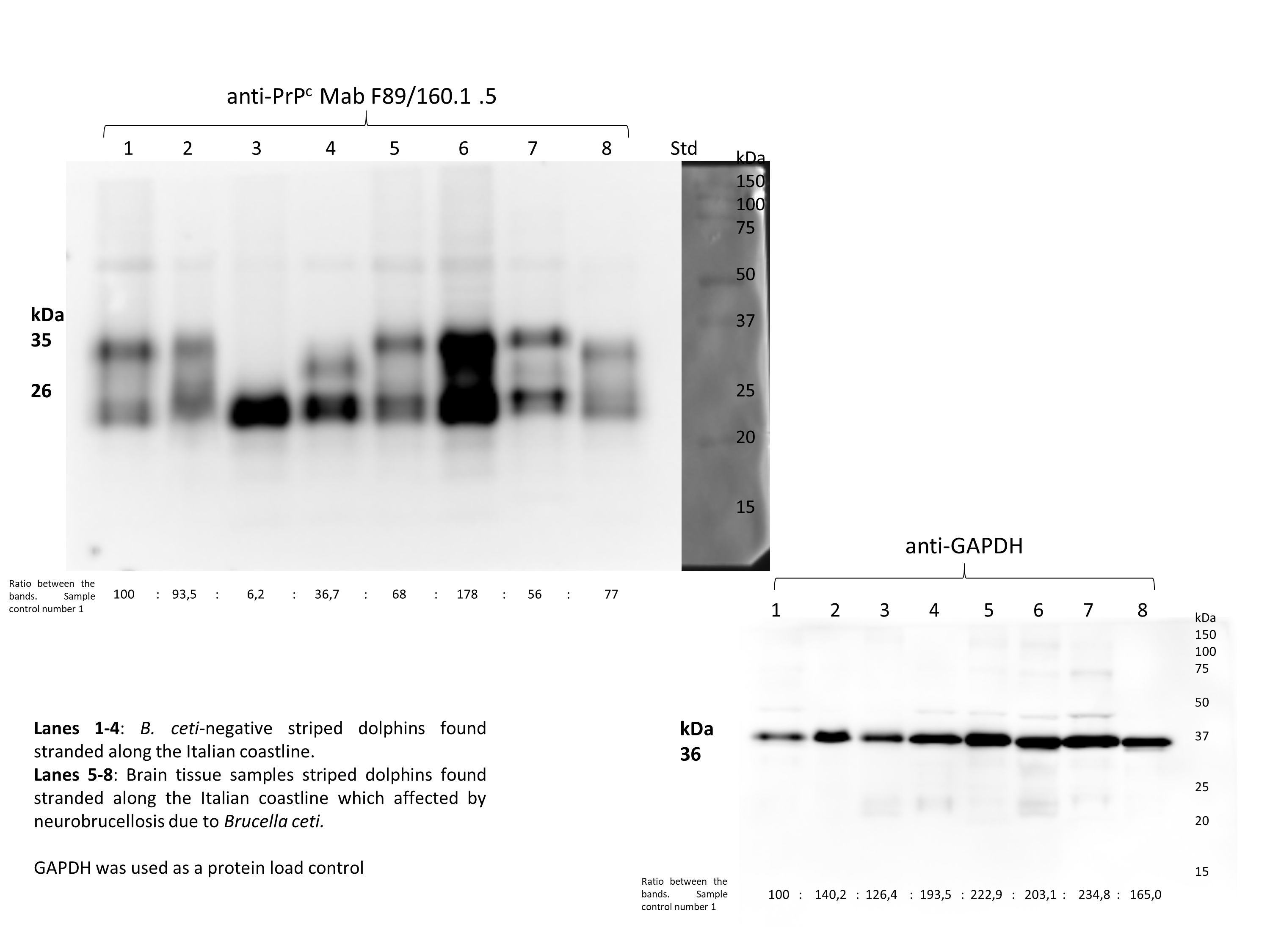

Supplement: Supplementary file 1 [file animals-12-01304-s001.zip › FigureS2.TIF]

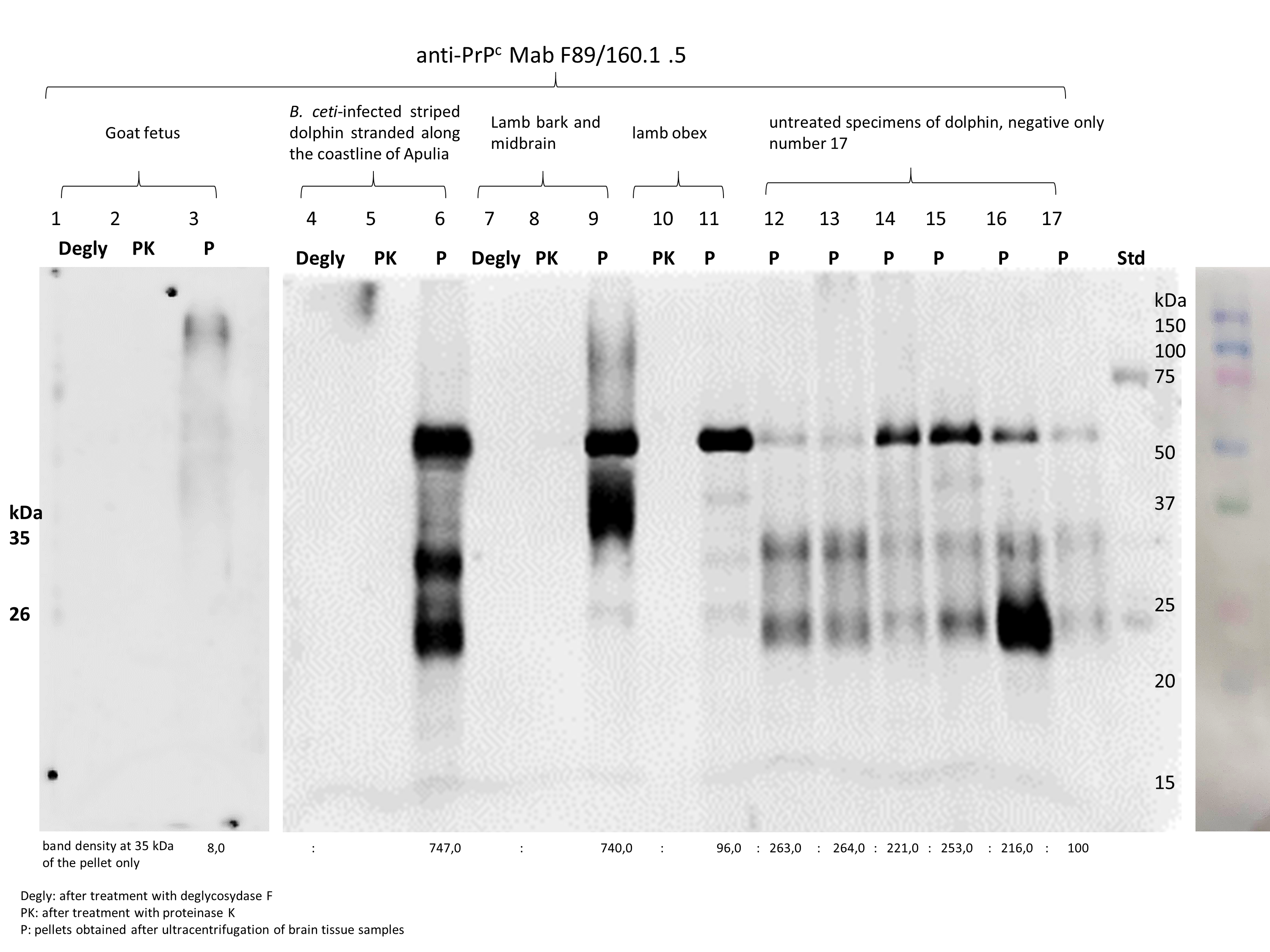

Supplement: Supplementary file 1 [file animals-12-01304-s001.zip › FigureS3.TIF]

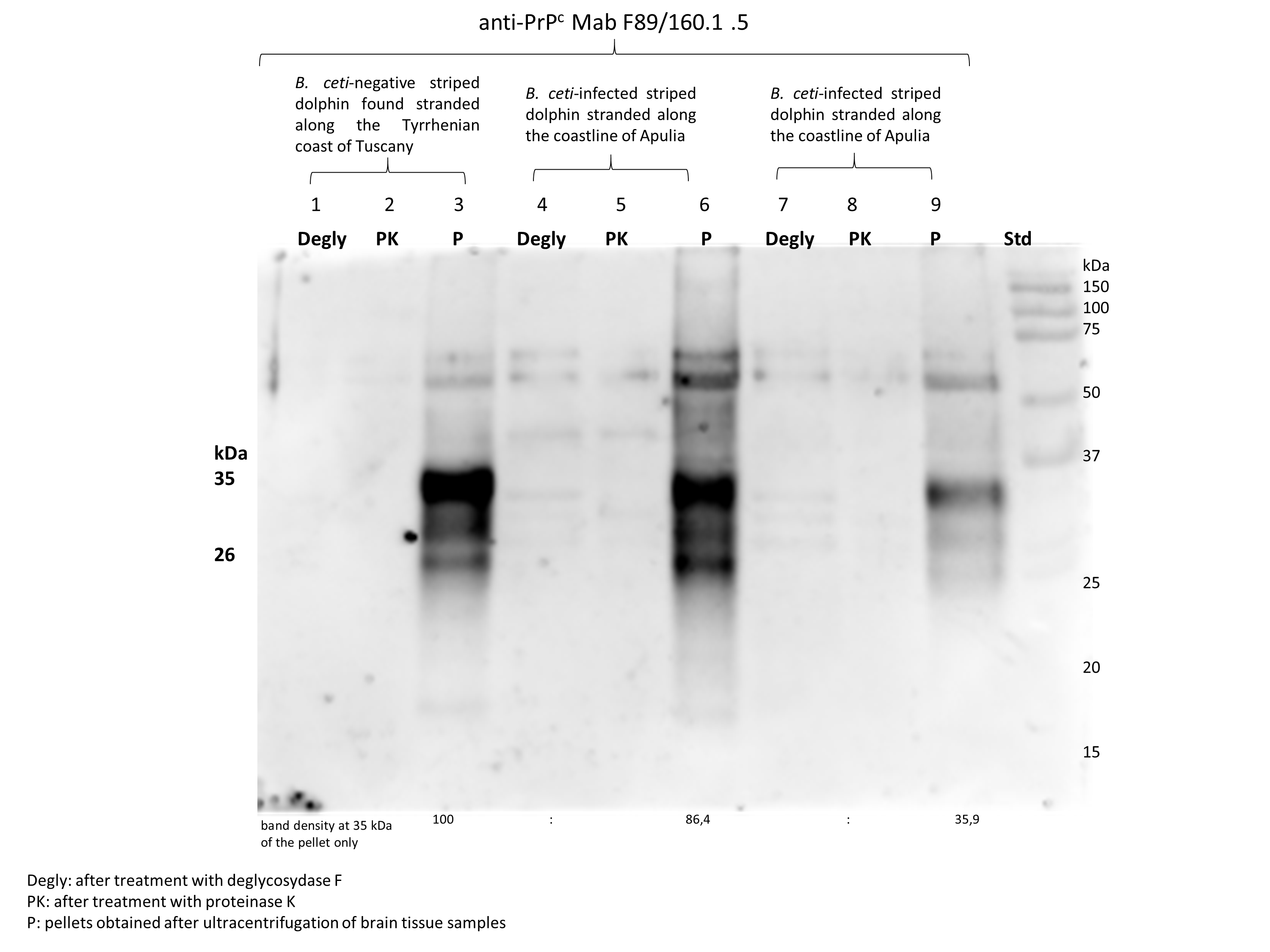

Supplement: Supplementary file 1 [file animals-12-01304-s001.zip › FigureS4a.TIF]

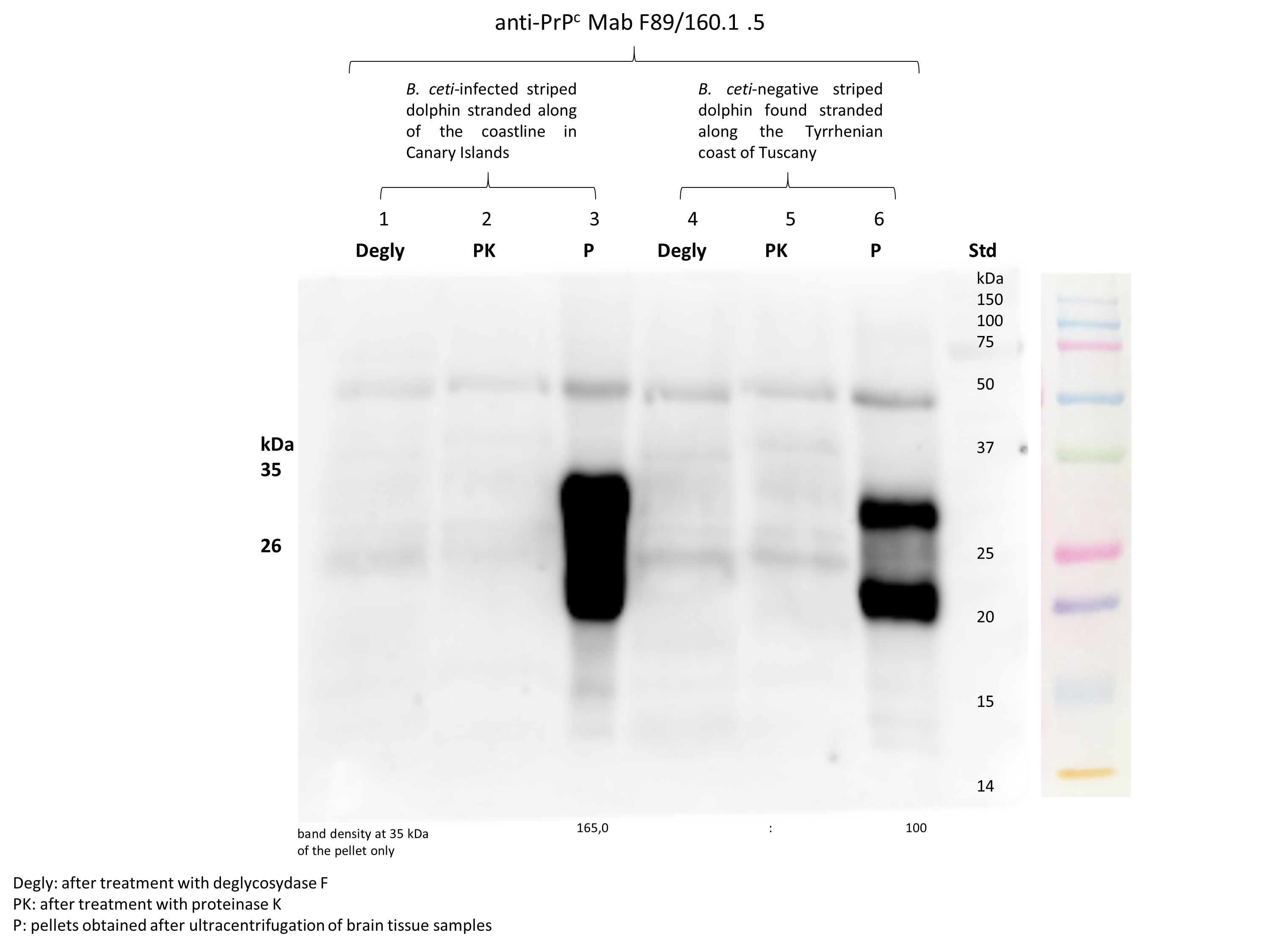

Supplement: Supplementary file 1 [file animals-12-01304-s001.zip › FigureS4b.TIF]
